# Modified Unipolar Hemiarthroplasty for the Treatment of Metastatic Lesions of Proximal Femur with Pathological Fractures: Case Series of Six Patients

**DOI:** 10.5704/MOJ.1911.004

**Published:** 2019-11

**Authors:** CY Lim, S Mat-Hassan, M Awang, MF MD-Ariff, MA Hau-Abdullah

**Affiliations:** Department of Orthopaedics and Traumatology, Hospital Kuala Lumpur, Kuala Lumpur, Malaysia; * Department of Orthopaedics, Hospital Raja Perempuan Zainab II, Kota Bharu, Malaysia

**Keywords:** proximal femur, metastatic lesions, pathological fractures, endoprosthesis, hemiarthroplasty

## Abstract

**Introduction:** Proximal femur resection and endoprosthetic reconstruction is the preferred treatment for extensive bony destruction and pathological fractures. Due to the relatively high cost of endoprosthesis, we adopted the modified unipolar hemiarthroplasty (MUH) for reconstruction when the mode of treatment was for palliation.

**Materials and Methods:** This is a retrospective case study of six patients, who had bone and multi-organs metastases with extensive proximal femur involvement with pathologic fractures who underwent resection and MUH reconstruction during the period 2013 to 2017. All patients were classified as Group B / C based on Scandinavian Sarcoma Group survival scoring, with estimated survival of maximum six months. The basic MUH construct consisted of Austin-Moore prosthesis which was secured to a Küntscher nail using cerclage wire and cemented into the femoral canal. Subsequently, the whole length of the prosthesis which remained outside the canal was coated with cement.

**Results:** The mean age was 61.8 years. The mean survival was 3.9 months, post-operation. There was no implant failure during patients’ life span; however, a third of the patients developed infection. Wheel chair ambulation was started immediately post-operation for all patients, and two patients progressed to walking frame ambulation. The total cost of each construct was below US$490 in comparison to long-stem hemiarthroplasty (roughly US$ 1700).

**Conclusion:** Our aim was to alleviate pain, facilitate rehabilitation, ease nursing care and improve quality of life for metastatic bone disease patients until end of life. MUH for the treatment of pathological fracture in proximal femoral metastases is a feasible palliative surgical modality in resource-limited settings.

## Introduction

The proximal femur is the long bone most commonly affected by metastatic disease^1, 2^. It is also a common site for pathological fracture because a significant force passed through this region^3, 4^. Impending and pathological fractures of the proximal femur can have a disastrous effect on the patient’s quality of life, resulting in severe pain and limitation in function and mobility^5^. The primary goals of surgical stabilisation of proximal femur metastatic lesions are to relieve pain, allow immediate return to function, improve the patient’s quality of life, facilitate medical treatment, and decrease the chance of subsequent surgical revision^5^. Surgical options that have emerged to achieve these goals include plate fixation, intramedullary fixation and endoprosthetic reconstruction. Traditionally, proximal femur resections are reconstructed with endoprosthesis^6^. However, due to the cost of endoprosthesis, a more affordable reconstruction method was devised which we termed “modified unipolar hemiarthroplasty (MUH)” and which was used when treatment was solely for palliation in patients with very unfavorable prognosis.

## Materials and Methods

This was a retrospective case series of six patients with metastatic disease of the proximal femur who underwent modified unipolar hemiarthroplasty (MUH) surgery between years 2013 to 2017, retrieved from our hospital’s operation record database. All data were obtained from medical records and radiological imaging, and no patient was specifically recalled for this study. As such, institutional review board approval was not required for this study. All six patients were diagnosed with metastatic bone disease before being subjected to the surgery. Prior to surgery, blood investigations and radiological investigations (Plain radiographs and magnetic resonance imaging (MRI) of the affected site, computed tomography (CT) scan of thorax, abdomen and pelvis for staging) were performed. The patients and their family were informed about the operation modifications, and written individual consent was obtained from them prior to surgery. We collected patients’ data on age, pre-operative blood counts, Charlson Comorbidity Index Score, modified Bauer Score, Scandinavian Sarcoma Group (SSG) Survival Scoring, operation time, estimated intra-operative blood loss, implant used, complications, histopathological examination (HPE) results, duration of post-operative stay and survival period after the surgery. Only patients with poor prognosis (Patients in Group B or C based on SSG survival scoring) were subjected to MUH reconstruction.

The proximal femur resections were performed using standardised protocol by the senior author and the orthopaedic oncology team. The patient was placed in a lateral position and a long lateral incision was made extending from 3 to 4cm proximal to the greater trochanter to the distal two thirds of the thigh. The rest of the proximal femur resection was carried out as described by Bickels *et al*^7^ after which, the wound was irrigated, followed by reaming of the femoral canal to the appropriate diameter. To permit an adequate cement mantle, the Küntscher nail with the diameter 2mm less than the last largest reamer size utilised was selected. In order to span the whole length of the femur, the nail length was determined by adding the resected specimen length and the reaming length. The appropriately size Austin-Moore hip prosthesis was chosen based on the femoral head measurement. The chosen prosthesis was utilised to test the “suction” fit of the head in the acetabulum. Full seating of the head was checked.

The preparation of the MUH was performed on a separate sterile operating trolley. The selected Austin-Moore hip prosthesis was secured to the Küntscher nail using cerclage wire. In order to increase the implant strength, we further improvised our modified unipolar hemiarthroplasty construct during the two more recent cases. The decisions to further modify the construct were made prior to surgery during pre-operative planning after we measured the size of the femoral canal. The patients and family were informed about the possibility of intra-operative additional modifications to the implants during pre-operative counselling. We added Rush rod for our fifth case. During our final case, we slotted a smaller diameter nail inside the bigger Küntscher nail and threaded the cerclage wire through both the Küntscher nail eyelets before securing the nails to the Austin-Moore hip prosthesis.

The femoral canal was again irrigated and dried. The soft-tissues, especially those which were near to the neurovascular structures, were protected and packed off with wet abdominal pads. As a guide to appropriate anteversion, marking was made on the anterior cortex of the femur as a guide with the aim to place the prosthesis in 10° to 15° anteversion. Bone cement was then mixed and allowed to cure to a doughy consistency before being injected into the femoral canal in a retrograde manner. The Küntscher nail end of the MUH was then inserted into the femoral canal and firmly held in place while waiting for the cement to harden.. Subsequently, another packet of bone cement was mixed, and moulded circumferentially along the whole length the MUH which remained outside of the femoral canal.

The remaining hip capsule was sutured tightly around the neck of the Austin-Moore prosthesis. Depending on whether the greater trochanter had been resected *en bloc* with the surgical specimen, the remaining abductor tendon or the residual fragment of the greater trochanter was anchored to the prosthesis through the eye of the Austin Moore prosthesis. Tenodesis of vastus lateralis to the abductor muscle fixation was subsequently performed. The wound closure and post-operative care were similar to patients undergoing proximal femur endoprosthetic replacement, including advice on hip dislocation precaution. We started our patient on wheel chair ambulation first followed by walking frame ambulation as tolerated, as opposed to immediate weight bearing ambulation, due to generally weaker condition of our patients.

The patients had been followed-up in the clinic at two weeks and one month after discharge from hospital, and subsequently followed-up three monthly until death. Mortality was tracked through medical records and telephone calls to the patients’ family when the patients did not turn up for the scheduled follow-up.

## Results

There were four male and two female patients with age range 56 to 73 years, (mean 61.8 years). Four patients had sustained pathological subtrochanteric fractures while two patients had pathological neck of femur fractures. One patient was known to have adenocarcinoma of the rectum with lung metastasis prior to the pathological subtrochanteric fracture. The other five patients had pathological proximal femur fracture as their first presentation prior to detection of primary malignancy. All patients were noted to have multiple organ metastases from CT scan prior to operation. The mean Charlson Comorbidity Index Score was 8.5 points (Range 7 to 11 points), while the mean modified Bauer Score was 1 (Range 0 to 2). Four patients scored one point based on SSG survival scoring system, which placed them under group B with estimated survival of three to six months. Two patients scored 0 point, with estimated survival of less than three months (Group C).

All patients underwent MUH palliative surgery. The mean estimated blood loss was 1433.3 mls (Range 400 to 4000mls). The operation time ranged from 150 minutes to 420 minutes. The total cost of each construct was below US$490. Two patients developed surgical site infections (33.3%); one patient was treated with intravenous antibiotics, while the other underwent surgical debridément.

Wheel chair ambulation was started almost immediately after surgery for all our patients. One patient managed to ambulate with walking frame on 6th post-operation day, while another patient was able to ambulate with walking frame few weeks after surgery. The final histopathological examination (HPE) of the resected specimens revealed metastatic lesions with primaries from the lung in three cases and one each from thyroid, kidney and rectum. All patients had passed away within 36 days to 11 months after operation with the mean of 3.9 months post-operation survival.

**Fig. 1:**
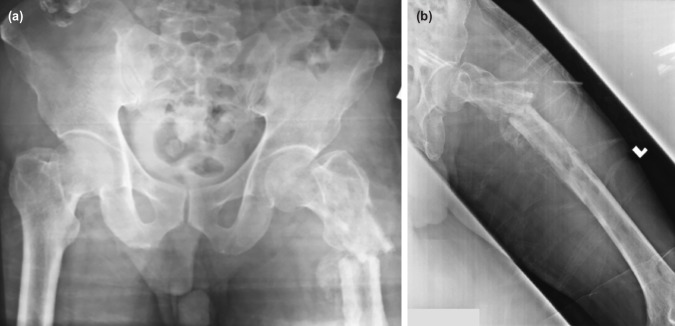
Mr. N, a 63-year old male, with left thigh pain after trivial fall. Radiographs (a) and (b) showing diffuse lytic lesion over the whole length of left femur with pathological subtrochanteric fracture.

**Fig. 2:**
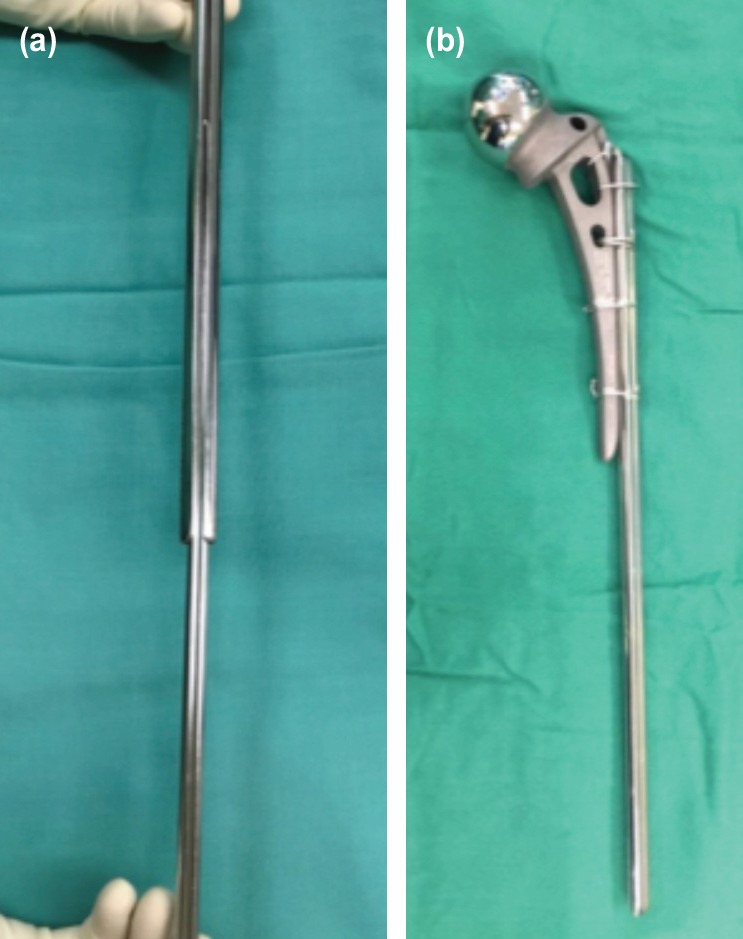
(a) The strength of the construct is further augmented by slotting in a smaller size Küntscher nail into the larger nail. (b) Modified unipolar hemiarthroplasty (MUH) constructed using Austin-Moore hip prosthesis secured to the Küntscher nail using cerclage wires.

**Fig. 3:**
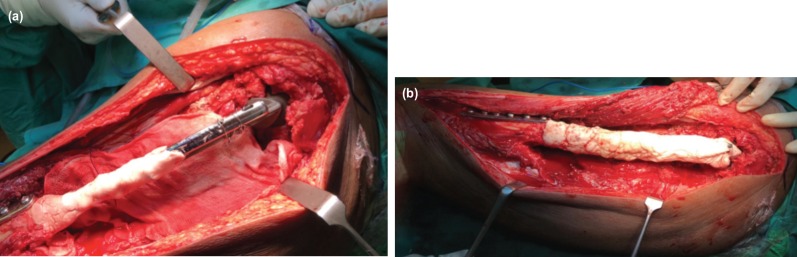
Intra-operative photographs (a) and (b) showing the remaining cement moulded circumferentially to coat the whole length of the prosthesis which remained outside of the canal. A 9-hole distal femur buttress plate with cement augmentation was used to address the lytic lesions which involved the shaft and distal femur.

**Fig. 4:**
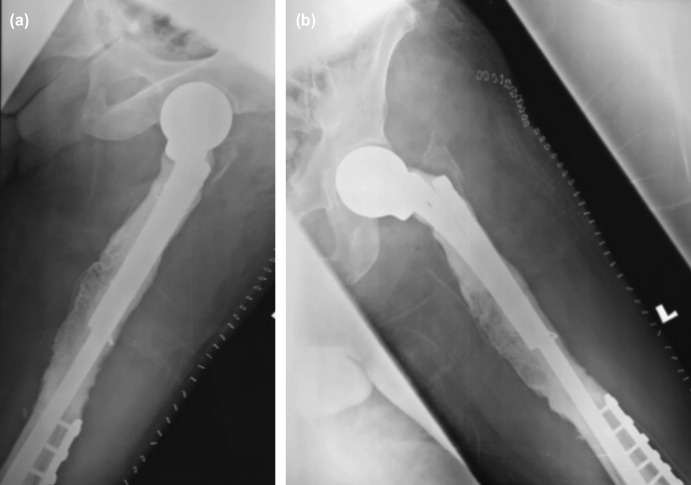
Post-operative radiographs (a) and (b) showing the modified unipolar hemiarthroplasty (MUH) construct. A 9-hole distal femur buttress plate with cement augmentation used to address the lytic lesions which involved the shaft and distal femur.

Table I summarises various parameters, including the patients’ condition prior to surgery, surgical information and post-operative outcome of all six patients. Figures 1-4 illustrate the case of pathological subtrochanteric fracture with metastatic lesion involving the entire length of femur in a 63-year old man with metastatic lung carcinoma.

**Table I:** Summary of parameters in the 6 patients with metastatic pathological fracture of proximal femur.

	Patient 1	Patient 2	Patient 3	Patient 4	Patient 5	Patient 6
Age (years old)	56	73	64	56	59	63
White cell count (X109/L)	8.6	12.58	7.39	20.83	9.66	8.05
Hematocrit (%)	35.8	40.5	35.1	41.5	40.7	30
Charlson Comorbidity Index score	7 points	10 points	11 points	7 points	8 points	8 points
Modified Bauer Score	1 point	0 point	2 points	0 point	1 point	0 point
Scandinavian Sarcoma Group (SSG) survival scoring	1 point (Group B)	0 point (Group C)	1 point (Group B)	0 point (Group C)	1 point (Group B)	1 point (Group B)
Metastatic sites from pre-operative staging	Lung, lymph nodes, adrenal & bone metastases	Lung, liver, adrenal and bone metastases	Lung, liver and bone metastases	Nodal, lung, adrenal and bone metastases.	Lungs and bone metastases	Lung, liver, adrenal, renal and bone metastases
Site of pathological fracture	Subtrochanteric	Neck of femur (and tibial lytic lesion)	Subtrochanteric	Neck of femur	Subtrochanteric	Subtrochanteric fracture and lesions involving whole femur
Operation time	150 minutes	265 minutes (2 procedures)	152 minutes	420 minutes	220 minutes	305 minutes (2 procedures)
Estimated blood loss	1500mls	400 mls	400 mls	800 mls	1500 mls	4000mls
Implant used to reconstruct the modified unipolar hemiarthroplasty (MUH)	Austin Moore prosthesis size 46mm Küntscher nail size 9mm X 320mm Cerclage wire 1.2mm X 3 Smartset MV Endurance® bone cement 2 packets	Austin Moore prosthesis size 42mm Küntscher nail size 12mm X 360mm Cerclage wire 1.2mm X 3 Simplex® P bone cement 3 packets (Combined with resection of tibia metastatic lesion and reconstruction procedure)	Austin Moore prosthesis size 47mm Küntscher nail size 12mm x 360mm Cerclage wire 1.2mm x 3 Simplex® P bone cement 3 packets	Austin Moore prosthesis size 47mm Küntscher nail size 12mm x 360mm Cerclage wire 1.2mm x 3 Simplex® P bone cement 3 packets	Austin Moore prosthesis size 41mm Küntscher nail size 11mm x 320mm Cerclage wire 1.2mm X 5 Rush rod X1 Simplex® P bone cement 4 packets	Austin Moore prosthesis size 50mm Küntscher nail size 14mm X 360mm Küntscher nail size 8mm x 60mm Cerclage wire 1.2mm X3 Simplex® P bone cement 5 packets 9-hole distal femur buttress plate
Post-operation hospital stay	11 days	16 days, transferred to nearby hospital	5 days	Succumbed to disease at day 36 post-operation	9 days	9 days
Histopathological examination (HPE) results	Metastatic anaplastic carcinoma of the thyroid	Metastatic adenocarcinoma, most likely from lungs	Metastatic carcinoma, most probably from renal cell carcinoma	Metastatic adenocarcinoma, most likely from lungs	Metastatic adenocarcinoma from the rectum	Metastatic adenocarcinoma, most likely primary from the lung
Complications from surgery	None	Prolonged ICU admission for 15 days	Surgical site infection	None	None	Anaemia induced acute coronary syndrome Surgical site infection
Survival duration post-operation	2 months	2 months	3 months	36 days	11 months	4 months

## Discussion

Pathological fracture of proximal femur causes pain and leads to deterioration of the quality of life. Furthermore, the patient with pathological fracture would be bed bound and at risk of developing potential complications of prolonged immobilisation, such as pressure ulcers, urinary tract infection, orthostatic pneumonia, deep vein thrombosis and potential pulmonary embolism. Thus, surgical management is indicated to improve quality of life, ease the nursing care and preserve mobility of these patients if they are fit to undergo surgical intervention after pre-operative assessment and optimisation of underlying medical conditions. A study of 5,716 patients undergoing musculoskeletal tumor surgery demonstrated that older age, male sex, blood transfusion, longer duration of anaesthesia, and higher Charlson Comorbidity Index Score were associated with postoperative complications^8^. The modified Bauer Score is commonly used to estimate life expectancy in patients with bone metastases^9, 10^ and score for our patients was 1 (Range 0 to 2), the treatment goal according to this scoring system was for short term palliation and supportive care. SSG Skeletal Metastases Registry is the world’s largest registry of surgically treated skeletal metastases and contains a total of 1195 skeletal metastases in 1107 patients^2^. The SSG survival scoring system offers a useful tool for estimating survival^2^. Based on this scoring system, all the patients in our case series had estimated survival of maximum six months, with a third had estimated survival of less than three months prior to surgery. In view of this, the mode of our MUH surgery was of palliative intent. We engaged both medical and anesthesiology teams as part of a multi-disciplinary approach to optimise our patients’ general medical conditions during the peri-operative period.

There are several reconstruction options for proximal femur metastasis; however, there are limited options available for pathological neck of femur fracture and peri-trochanteric femur with massive bony destruction where implant failure in a major concern. Janssen *et al* reported that fixation failure was most common after open reduction and internal fixation

(13% versus 3.0%) than intramedullary nailing and none after endoprosthetic reconstruction^6^. Long stem cemented hemiarthroplasty is another reasonable treatment option for patients with proximal femur metastasis. Peterson *et al* reported fair levels of function among 21 patients treated with long stem cemented hemiarthroplasty in 22 limbs for metastases^11^. However, Maheshwari *et al* reported 6.6% stem fracture rate among 61 long stem cemented hemiarthroplasty performed for oncologic indications^12^. The cost of long stem bipolar hemiarthroplasty in our setting is around USD 1,700. Endoprosthetic reconstruction is the treatment of choice in ideal situation where implant cost is not an issue. However, most of the patients in our government hospital setting cannot afford tumor endoprosthesis, which costs more than USD 6,000.

Furthermore, the patients in our case series had short estimated survival period prior to surgery, the mode of treatment for all six patients were palliative intent due to multiple metastases. Thus, it was not reasonable to burden the family or the government healthcare trust fund with high implant cost if we could offer a cheap alternative reconstruction method which could achieve the same goal. The palliative surgeries were aimed to alleviate pain, improve mobility, facilitate rehabilitation, ease the nursing care and improve quality of life for these patients until the end of life. We devised MUH to reconstruct the proximal femur post- resection specifically for metastatic bone disease patients with poor prognosis due to financial consideration and the construct lasted throughout patients’ lifetime and there was no implant failure in all six patients. We did not try to equate MUH to proximal femur endoprosthesis as the modified construct strength will definitely be weaker especially at the junction where the Austin-Moore prosthesis is held with cerclage wires to the Küntscher nail. The benefit of this modified construct is the low cost, considering that the cost of our MUH was more than ten times cheaper than the proximal femur oncology prosthesis and about a third cheaper than the long stem bipolar hemiarthroplasty in our setting. All the components used to assemble the MUH were readily available in the Malaysian government hospital orthopaedic implants stock. Thus, there was no delay in surgery due to waiting time for implant financial aid approval and the patient could go for surgery once evaluated and suitable.

There are several shortcomings of our modified prosthesis case series. Firstly, this is a small case series in a single institution. There is no control group which limits the meaningful comparison of surgical outcomes with other reconstruction methods. Secondly, we did not have mechanical testing performed on our MUH. However, all the components used to construct the MUH were time-tested standardised orthopaedic implants such as Austin Moore prosthesis, Küntscher nail and cerclage wire. Austin Moore prosthesis was chosen because of the available two fenestrations which allowed tying of the Küntscher nail to the unipolar hemiarthroplasty using cerclage wires. Besides that, the eye for removal on the Austin Moore prosthesis could be used to anchor the remaining abductor tendon or the residual fragment of the greater trochanter. The drawback for this MUH is that there is only one place to anchor the soft tissues to the prosthesis in comparison to conventional proximal femur endoprosthesis where several holes are available for this purpose.

Prosthetic replacement has a lower risk of revision surgery compared to internal fixation in the treatment of pathological fractures or impending fractures of the proximal femur^13^. At present, prosthetic dislocation is the most common complication observed in patients managed by prosthesis replacement of the proximal femur^13^. We repaired the remaining hip capsule tightly around the neck of the Austin Moore prosthesis. There was no dislocation in all six patients; however, we had a very small number of patients in this series to make definitive conclusions on potential implant-related complications.

Two patients developed surgical site infections (33.3%) within the first month after operation, one treated with intravenous antibiotics, and the other underwent surgical debridément. Janssen *et al*^6^ in their retrospective study on complications after management of proximal femoral metastases on 417 patients, concluded that deep infections (8.6%) were most common following 70 endoprosthetic reconstructions and occurred within the first month after surgical treatment. Harvey *et al* reported ten infections (9%) among 113 endoprosthetic replacement cases performed for proximal femur metastases^5^. However, these comparisons were not robust as the pre-operative conditions of the patients in these studies were not similar to our case series. We did not find any article which specifically addresses the issue of palliative surgery in patients with projected survival of less than six months. The fairly high infection rate in our case series could be due to the poor general pre-operative medical conditions of our patients. Nevertheless, good peri-operative wound care is very important to reduce the surgical site infection rate, and we would strive to reduce our infection rate in our future cases. This MUH modification is still a useful reconstruction option despite not having been mechanically tested previously and a third of our patients developing surgical site infection, and it may be a feasible palliative alternative reconstruction in resource-limited settings.

## Conclusion

Modified hemiarthroplasty for the treatment of pathological fracture in proximal femoral metastases in resource-limited settings, is a feasible palliative surgical treatment modality with the aims of restoring useful function, reducing pain and providing reasonably good quality of life for patients during their projected short survival period, without the need for revision surgery.
